# Microtubule nucleation by γ-tubulin complexes and beyond

**DOI:** 10.1042/EBC20180028

**Published:** 2018-10-12

**Authors:** Corinne A. Tovey, Paul T. Conduit

**Affiliations:** Department of Zoology, University of Cambridge, Downing Street, Cambridge CB2 3EJ, U.K.

**Keywords:** centrosome, gamma-tubulin ring complex, g-TuRC, microtubule, MTOC

## Abstract

In this short review, we give an overview of microtubule nucleation within cells. It is nearly 30 years since the discovery of γ-tubulin, a member of the tubulin superfamily essential for proper microtubule nucleation in all eukaryotes. γ-tubulin associates with other proteins to form multiprotein γ-tubulin ring complexes (γ-TuRCs) that template and catalyse the otherwise kinetically unfavourable assembly of microtubule filaments. These filaments can be dynamic or stable and they perform diverse functions, such as chromosome separation during mitosis and intracellular transport in neurons. The field has come a long way in understanding γ-TuRC biology but several important and unanswered questions remain, and we are still far from understanding the regulation of microtubule nucleation in a multicellular context. Here, we review the current literature on γ-TuRC assembly, recruitment, and activation and discuss the potential importance of γ-TuRC heterogeneity, the role of non-γ-TuRC proteins in microtubule nucleation, and whether γ-TuRCs could serve as good drug targets for cancer therapy.

## Introduction

**Figure 1:**
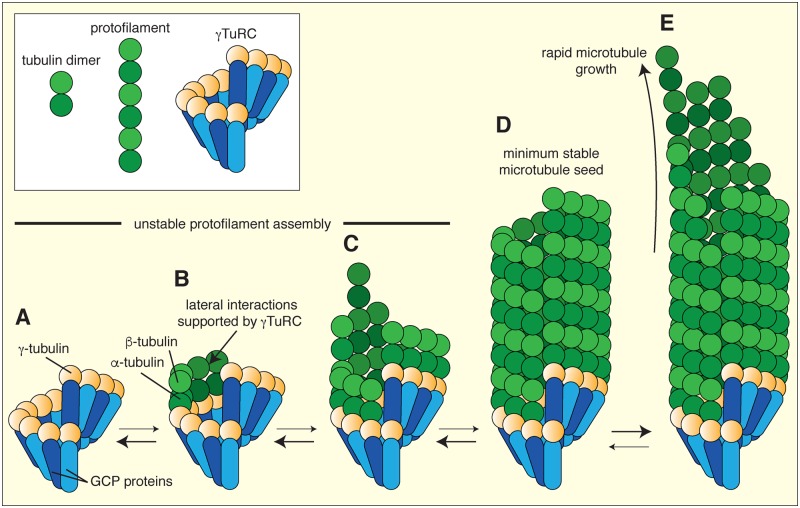
Templated microtubule nucleation (**A**) γ-tubulin molecules (yellow) within the γ-TuRC are positioned in a single-turn helix via their binding to GCP proteins (blue). (**B**) γ-tubulin molecules bind to incoming α/β-tubulin dimers from the cytosol and this is thought to promote the lateral interaction between the α/β-tubulin dimers as they grow into protofilaments (a protofilament is a single end-to-end chain of tubulin dimers). (**C**) Microtubule assembly progresses slowly through an unstable stage where disassembly is more likely than continued assembly (as indicated by the thickness of the two-way arrows). (**D**) Assembly is thought to reach a stable stage, where a microtubule seed containing sufficient tubulin dimers has formed (although the size of this stable seed remains unclear). (**E**) Once the stable seed has formed, microtubule polymerisation is favoured and can progress rapidly. Abbreviation: GCP, γ-tubulin complex protein.

Microtubules are polarised polymers involved in a wide range of cellular processes including chromosome separation, intracellular transport, organelle positioning, cell–cell signalling and cell motility [1]. Tight spatial and temporal regulation of the formation, organisation, and dynamic behaviour of the microtubule cytoskeleton is extremely important. Consequently, cells have developed complex mechanisms to regulate microtubule nucleation, polymerisation and catastrophe, severing, stabilisation and transportation. Collectively, these processes establish diverse microtubule networks with highly specialised functions in different cells or at different times during the cell cycle. Multiprotein γ-tubulin ring complexes (γ-TuRCs) template microtubule nucleation within cells (Figure 1) [2–5]. The γ-tubulin molecules within this complex are positioned in a single-turn helical pattern [6] and the end-on interactions between γ-tubulin and α/β-tubulin dimers most likely help to support the lateral association between α/β-tubulin dimers during their assembly into protofilaments; this is thought to promote the otherwise kinetically unfavourable formation of a short tubular structure (the microtubule seed), which, once beyond a certain size threshold, can rapidly polymerise into a microtubule filament ([3,7]; Figure 1). γ-TuRCs appear to regulate microtubule polarity, as they are always positioned at the α-tubulin-containing ‘minus end’ with the highly dynamic β-tubulin-exposed plus end extending outwards (Figure 1). Microtubule nucleation must be highly regulated in space and time to ensure the correct formation of microtubule networks [8,9]. Thus, γ-TuRCs are normally activated only once recruited to specific sites within the cell known as microtubule organising centres (MTOCs). How γ-TuRCs are assembled, recruited and activated are still key areas of interest in the field.

## γ-TuRC composition

### The γ-tubulin small complex

**Figure 2:**
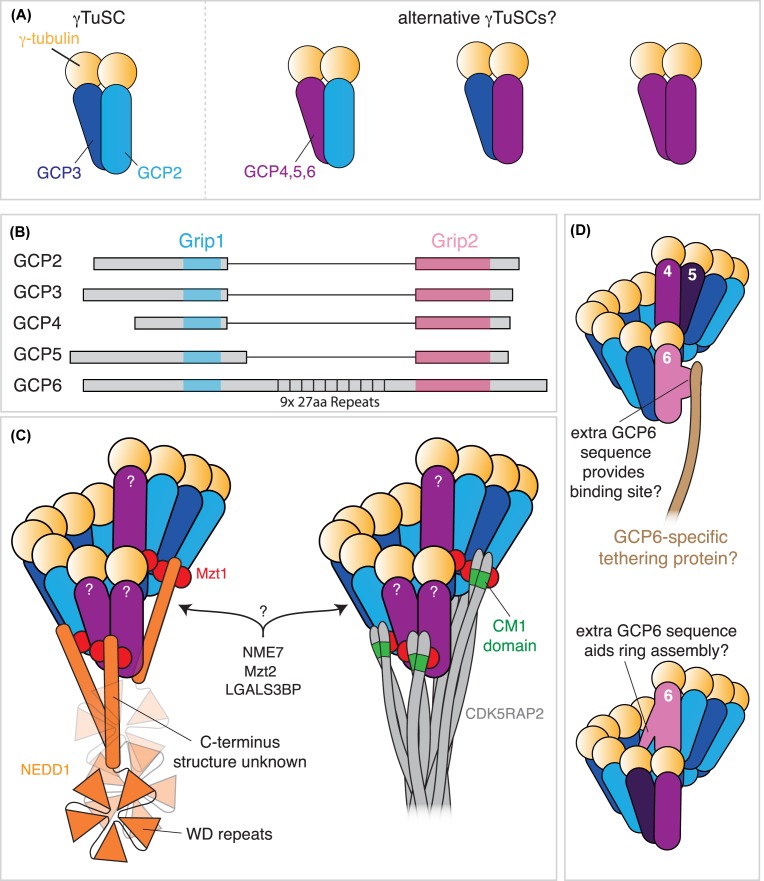
Non-core γ-TuRC components (**A**) The canonical γ-TuSC comprises two molecules of γ-tubulin and one each of GCP2 and GCP3, but alternative γ-TuSCs may exist in which GCP2, GCP3, or both are replaced by either GCP4, 5, or 6. (**B**) GCP2–6 all contain a Grip1 and a Grip2 domain. The Grip1 domain mediates interactions between GCP proteins, while the Grip2 domain mediates interactions with γ-tubulin. In addition, GCP6 contains an expanded central region that includes nine 27-aa repeats of unknown function. (**C**) GCP4, 5 and 6 (depicted here in purple) are predicted to replace some of the GCP2 and GCP3 molecules within the ring, but their exact positions remain unknown. MZT1 binds to the N-terminal regions of GCP proteins and acts as an adapter protein for the binding of the tethering protein NEDD1 (left) and tethering proteins that contain an N-terminal CM1-domain, such as CDK5RAP2 (right). NEDD1 contains putative WD40 repeats that form a β-propeller structure known to mediate protein–protein interactions (presumably with proteins at MTOCs). The structure of the C-terminus of NEDD1 is currently unknown but is required for binding to the γ-TuRC. The positions of NME7 kinase, MZT2 and LGALS3BP remain unknown. (**D**) The function of the extra sequence in GCP6 is unknown, but may provide a binding site for a GCP6-specific tethering protein (top) or may function in ring assembly by forming interactions with other γ-TuRC components (bottom). Abbreviations: CM1, centrosomin motif 1; MZT1, MOZART1; MZT2, MOZART 2.

The core subunit of the γ-TuRC is the γ-Tubulin Small Complex (γ-TuSC), a heterotetramer of ~300 kDa containing two molecules of γ-tubulin and one each of γ-tubulin complex protein 2 (GCP2) and GCP3 [10–14] (Figure 2A). In budding yeast, seven γ-TuSCs form a single-turn helix template with approximately the same pitch and diameter as a microtubule [6]. A similar process likely occurs in higher eukaryotes, but other types of GCP molecules are predicted to replace some of the GCP2/3 molecules within the ring [15] (see below). GCP2 and GCP3 are conserved in all eukaryotes and are essential for cell viability in all organisms in which they have been studied [10,12,16–22]. There is even some degree of functional conservation between species, as the homologues of GCP2 and GCP3 in fission yeast can be replaced to some extent by their human or budding yeast counterparts [23].

### GCP proteins and the γ-TuRC

Many species possess three additional GCP proteins, GCP4, 5 and 6 (Table 1), which share sequence and structural similarity with GCP2 and GCP3 [5,14,24,25]. GCP proteins have species-specific names, so for simplicity we will use the human nomenclature for all species. The structural similarity of GCP4, 5 and 6 with GCP2/3, together with their low stoichiometry within the γ-TuRC [25–27], suggests that GCP4, 5 and 6 replace some of the GCP2/3 molecules in the γ-TuRC ring ([3,5,24]; Table 1 and Figure 2A). This hypothesis is supported by the co-fractionation of GCP4 and GCP5 with the γ-TuSC [28] and domain swapping and FRET-based interaction experiments [29]. GCP2–6 possess a Grip1 and a Grip2 domain (Figure 2B); the N-terminal Grip1 domain appears to mediate lateral interactions between GCP proteins, while the C-terminal Grip2 domain associates with γ-tubulin [21,22,30,31] and can be swapped between GCP proteins [29]. Although the precise function and position of GCP4, 5 and 6 remain unclear (Figure 2C), their removal in different systems reduces γ-TuRC abundance in cytosolic extracts [19,28,32,33], suggesting a role in γ-TuRC assembly. Charged regions in GCP4 may prevent bidirectional lateral contacts with other GCP proteins, suggesting that GCP4 is involved in ring initiation or termination [15]. Nevertheless, unlike GCP2 and 3, GCP4, 5 and 6 are not essential for viability in *Drosophila*, fission yeast or *Aspergillus* [19,32,34,35], showing that proper assembly of γ-TuRCs (at least in the cytosol) is not required for a large proportion of γ-TuRC function. This is most likely because γ-TuRCs can assemble in the absence of GCP4, 5 and 6 at MTOCs (as suggested in [32]), similar to the natural situation in yeast cells (see below). One key function of GCP4, 5 and 6 in higher eukaryotes may, therefore, be to provide greater γ-TuRC diversity in the presence of a wider range of MTOCs (suggested in [5]). For example, GCP6 is involved in the localisation of γ-TuRCs to keratin fibres in epithelial cells [36], and GCP4 and GCP6 are required in both male and female germlines in *Drosophila* [32,35,37,38]. Very little is known about the additional sequences found in GCP5 and GCP6 (which, in GCP6, contains nine 27-aa repeats) and it is possible that these regions form important interactions either within the γ-TuRC structure or with tethering or modifying proteins at MTOCs (Figure 2D). Elucidating exactly how the extra GCP proteins integrate into γ-TuRCs will shed light on the relevance of these non-essential proteins.

**Table 1:** Orthologues of proteins involved in microtubule nucleation in selected species

Category	*Homo sapiens*	*Drosophila melanogaster*	*Arabidopsis thaliana*	*Caenorhabditis elegans*	*Candida albicans*	*Aspergillus nidulans*	*Schizosaccharomyces pombe*	*Saccharomyces cerevisiae*
γ-TuSC	γ-tubulin	γ-tubulin 23C/37C	TUBG1/2	Tbg1	Tub1	mipA	Tug1/Tubg1	Tub4
	GCP2	Grip84	Spc97p/GCP2	Grip1/Gip1	Spc97	GCP2/B	Alp4	Spc97
	GCP3	Grip91	Spc98p/GCP3	Grip2/Gip2	Spc98	GCP3/C	Alp6	Spc98
γ-TuRC GCPs	GCP4	Grip75	GCP4	?	-	GCP4/D	Gfh1	-
	GCP5	Grip128	GCP5	?	-	GCP5/E	Mod21	-
	GCP6	Grip163	GCP6	?	-	GCP6/F	Alp16	-
γ-TuRC other	NEDD1/GCP-WD	Grip71	Nedd1	?	-	-	-	-
	MOZART1	Mozart1	GIP1a/b	Mzt1	Mzt1	Mzt1/MztA	Mzt1	-
	MOZART2A	-	-	?	-	-	-	-
	MOZART2B							
	NME7	Nmdyn-D7	?	-	?	?	?	?
	LGALS3BP	?	?	?	?	?	?	?
CM1-domain γ-TuRC tethering	CDK5RAP2, Myomegalin, Pericentrin	Cnn, (Plp - no CM1 domain)	?	?	Spc110, Spc72	PcpA, ApsB	Pcp1, Mto1/Mto2	Spc110, Spc72
γ-TuRC- independent microtubule nucleators	chTog/CKAP5	Msps	?	Zyg9	?	?	Alp14	Stu2
	TPX2	Mei38	Tpx2	Tpxl-1	-	-	-	-

The table shows orthologues of γ-TuRC proteins, CM1-domain-containing γ-TuRC tethering proteins and γ-TuRC-independent proteins involved in microtubule nucleation across a selection of species, as indicated. ‘-’ refers to cases where attempts in the literature have failed to identify an orthologue and ‘?’ refers to cases where we are unaware of any attempts to identify an orthologue. Abbreviation: CM1, centrosomin motif 1.

### Other γ-TuRC proteins

In organisms other than budding yeast, γ-TuRCs contain additional proteins. The first was discovered in *Drosophila* and named Dgrip71WD [39] (now known simply as Grip71). Despite its name, Grip71 lacks Grip domains and instead contains N-terminal WD40 repeats (predicted to form a β-propeller structure that mediates protein–protein interactions) and a C-terminal domain with unknown structure required for Grip71’s association with the γ-TuRC (Figure 2C) [40–42]. Grip71 and its mammalian homologues (NEDD1, alternatively named as GCP-WD) have been extensively studied [32,39–56], collectively showing that these proteins are dispensable for γ-TuRC assembly and instead function in γ-TuRC recruitment to different MTOCs.

More recently, additional components of the γ-TuRC were identified: MOZART1 (MZT1), MOZART2 (MZT2), LGALS3BP and the kinase NME7 ( [26,27,57–59]; Table 1 and Figure 2C). Of these, MZT1 is the most widely conserved (Table 1) and has been the most extensively studied [28,38,58,60–67]. MZT1 homologues are small (~8.5 kDa) and comprise just three α-helices [65,66]; studies in plants, yeast and cultured human cells have shown that MZT1 interacts directly with the N-terminal region of GCP proteins (i.e. at the base of the γ-TuRC) [28,60–65] and that they regulate the interaction between the γ-TuRC and γ-TuRC-tethering proteins [28,64]. In all three systems, removal or knockdown of MZT1 impairs γ-TuRC recruitment to various MTOCs and cells fail in mitosis [28,58,60–64,66,67]. Surprisingly, however, MZT1 is not essential in *Drosophila* and is expressed only in the testes, where it is important for γ-TuRC recruitment to basal bodies, but not to mitochondria, in developing sperm cells [38]. It remains to be tested whether MZT1 also defines specific subsets of γ-TuRCs in other multicellular animal systems, and what impact this might have on γ-TuRC recruitment and function. Although MZT1 is clearly involved in γ-TuRC recruitment in cells where it is expressed, there are conflicting reports regarding a possible role for MZT1 in γ-TuRC assembly [28,64].

Less is known about the other newly discovered γ-TuRC proteins. MOZART2A and 2B are found only in deuterostomes; they have no sequence relation to MZT1 but are also small (~16.5 kDa) and are involved in γ-TuRC recruitment, although potentially only during interphase [26]. The kinase NME7 is required for efficient nucleation from centrosomes in human cells and increases the *in vitro* nucleation activity of purified human γ-TuRCs [68]. This increase, however, is relatively small and so other mechanisms may work synergistically with NME7 phosphorylation. Whether NME7 kinases function at γ-TuRCs in other systems remains to be tested. No study has yet investigated a potential role for LGALS3BP at γ-TuRCs, but its expression levels affect centrosome number and structure [59]. In summary, more work is needed before we have a full understanding of these additional γ-TuRC components.

## γ-TuRC assembly, recruitment and activation

### Assembly of the γ-TuRC ring

In the past, different models of γ-tubulin complex-mediated microtubule nucleation (template compared with protofilament) had been proposed [69,70]. The consensus now is that the template model is correct and that microtubule nucleation is catalysed after multiple γ-TuSCs assemble, often with other proteins, into ring-like structures. These structures can be regarded as γ-TuRCs, regardless of whether they contain γ-TuRC-specific components. One key question is how γ-TuRCs assemble. This is complex because assembly may occur in a cell- or MTOC-specific manner. In higher eukaryotes, such as humans, *Xenopus* and *Drosophila*, γ-TuRCs can assemble in the cytosol before being recruited to an MTOC. This cytosolic assembly depends, to potentially differing degrees, on GCP4, 5 and 6 [28,29,32,33,35] and, in some cell types, on Mzt1 [64]. In fission yeast, despite the presence of a Mzt1 homologue and GCP4, 5 and 6 homologues, γ-TuRCs are largely absent from the cytosol [34] and so presumably form specifically at MTOCs; here, assembly is catalysed by the binding of the Mto1/Mto2 complex (see below) [71]. Similarly, in budding yeast, which contains only γ-tubulin, GCP2 and GCP3, γ-TuRCs are absent from the cytosol and assembly occurs exclusively at Spindle Pole Bodies (SPBs, the yeast equivalent of centrosomes), stimulated by the binding of Spc110 (see below) [6]. Thus, at least two classes of γ-TuRC assembly exist: cytosolic assembly and MTOC-specific assembly, and it remains possible that both classes coexist within the same species or cell type.

### Recruitment to MTOCs

γ-TuRCs can be recruited to different MTOCs such as the centrosome, Golgi, nuclear envelope, cell cortex, mitochondria, and even the sides of pre-existing microtubules. This depends on the organism, cell type and cell cycle stage, and needs to be tightly regulated to ensure correct microtubule network formation. Consequently, there are a variety of proteins that can recruit γ-TuRCs to different MTOCs (that we term γ-TuRC-tethering proteins), including yeast Spc110, Spc72, Mto1 and Pcp1, *Drosophila* Cnn and Grip71 and mammalian CDK5RAP2, Myomegalin, Pericentrin and NEDD1. γ-TuRCs can also be recruited to different MTOCs by the same tethering protein. For example, NEDD1 recruits γ-TuRCs both to centrosomes and to the sides of pre-existing microtubules (via the multiprotein Augmin/HAUS complex) in human cells [40,41,46,72–75], and, while the major isoform of *Drosophila* Cnn recruits γ-TuRCs to centrosomes in dividing cells [76–80], testes-specific isoforms of Cnn recruit γ-TuRCs to mitochondria in sperm cells [81]. There are also differences between species because the *Drosophila* homologue of NEDD1, Grip71, is important for γ-TuRC recruitment to spindles (via Augmin) but not to centrosomes [32,47,82]. Most γ-TuRC-tethering proteins (except the Grip71/NEDD1 homologues) contain an N-terminal centrosomin motif 1 (CM1) domain of ~60 amino acids [83,84], which is necessary for binding and recruiting γ-TuRCs to MTOCs [27,64,76,85–87]. Evidence from the structure of Spc110-bound γ-TuRCs suggests the CM1 domain binds directly to the ring of GCP proteins [6], although this remains unproven. The CM1 domain is also necessary for γ-TuRC assembly in yeast [71,87] and for ectopic activation of γ-TuRCs in human and fission yeast cells [27,71]. Thus, the CM1 domain is capable of linking the processes of γ-TuRC recruitment, assembly, and activation.

Given the role of the CM1 domain in γ-TuRC activation (see also below), it seems sensible that CM1-domain proteins bind γ-TuRCs only at MTOCs, which presumably explains why endogenous CM1-domain proteins do not readily co-purify with γ-TuRCs from cytosolic extracts [14,25,39,58]. In contrast, Grip71/NEDD1 homologues do co-purify with γ-TuRCs and so do bind γ-TuRCs in the cytosol [39–41]. The reason for this difference is unclear, especially as Grip71 and NEDD1 are not required for cytosolic γ-TuRC assembly [32,40,41]. Intriguingly, the binding of CM1-domain proteins and Grip71/NEDD1 homologues is mutually exclusive, at least in some cell types [27,49], suggesting that they bind to the γ-TuRC in a similar region. It is therefore possible that Grip71/NEDD1 homologues bound to γ-TuRCs mask the binding of CM1-domain proteins in the cytosol in order to prevent premature γ-TuRC activation. Alternatively, the CM1-domain proteins may be unable to bind γ-TuRCs in the cytosol until they undergo MTOC-specific post-translational modifications. Importantly, both possibilities would help avoid the premature activation of γ-TuRCs. Moreover, it may be important to regulate closely which tethering protein binds the γ-TuRC, as NEDD1-bound γ-TuRCs serve to anchor microtubules while CDK5RAP2-bound γ-TuRCs nucleate microtubules in mouse keratinocytes [49]. It will be important in future to understand more about how the binding of different γ-TuRC-tethering proteins can regulate γ-TuRC recruitment and function.

### Regulation by phosphorylation and isoform expression

Many phosphorylation sites have been identified in various γ-TuRC components [36,88–95] and γ-TuRC tethering proteins, including Spc110 [84,91,96–100], NEDD1 [41,46,50–54,94,101], Grip71 [95], Pericentrin [102,103], CDK5RAP2 [94,103], Cnn [95,104–107] and Mto2 [108]. Several of these sites have been characterised and have been shown to play roles in γ-TuRC assembly, recruitment or activation, including at specific MTOCs. For example, phosphorylation of Ser^405^ in NEDD1 is required for chromosomal microtubule nucleation, but not centrosomal nucleation, in *Xenopus* egg extracts [50], while other phosphorylation sites in NEDD1 regulate its binding to the γ-TuRC [51,54]. Phosphorylation can also negatively regulate γ-TuRC recruitment and activity, as phosphorylation of GCP6 by CDK1 inhibits its binding to intermediate filaments in cultured human epithelial cells [36], and hyperphosphorylation of Mto2 in fission yeast during mitosis leads to the disassembly of the Mto1/Mto2 complex and subsequent inactivation of γ-TuRCs at non-SPB sites [108]. It is now important to try and understand exactly what effects these, and other, phosphorylation events have on the γ-TuRC to induce recruitment, activity or assembly, for example by inducing structural changes in the γ-TuRC. It will also be important to understand how phosphorylation can finely tune nucleation events in a cell- and MTOC-specific manner.

As mentioned above, the γ-TuRC-tethering proteins that contain a CM1 domain typically associate with γ-TuRCs only at MTOCs, and phosphorylation is a plausible mechanism to control this. For example, phosphorylation of Spc110 regulates its binding to γ-TuRCs to some degree [84] (although Spc110 oligomerisation is clearly also important [87]). Whether phosphorylation regulates the binding of other CM1-domain proteins remains unclear, but there is circumstantial evidence that this occurs in *Drosophila. Drosophila* Cnn is phosphorylated specifically at centrosomes [104] where it is important for γ-TuRC recruitment [76–80], and unphosphorylated Cnn does not associate with γ-TuRCs in *in vitro* binding assays [38]. How phosphorylation might regulate binding is unclear; it has been suggested that the extreme N-terminal region of Cnn folds back and inhibits the CM1 domain, as this N-terminal region is absent from testes-specific isoforms of Cnn that are able to bind γ-TuRCs *in vitro* [38]. Thus, the addition of negatively charged phosphate groups may help expose the CM1 domain to allow γ-TuRC binding. A similar region in yeast Spc110 could also be regulatory, as it is absent from the structure of Spc110-bound γ-TuRCs [6] and it is not essential for γ-TuRC binding [87]. This suggests that similar regulatory regions may exist in other CM1-domain proteins, but more work is needed to test these models and reveal any conservation across species. Given the recent findings that different Cnn isoforms bind differently to γ-TuRCs [38] and recruit γ-TuRCs to different MTOCs [81], it will be important to explore potential isoform differences in other γ-TuRC-tethering proteins and in Cnn homologues in different species, particularly as different CDK5RAP2 isoforms do exist [109–111]. It is likely that a combination of phosphorylation and isoform differences contributes to the tight spatiotemporal regulation of γ-TuRC recruitment and activation.

### Activation of γ-TuRCs

The assembly of the γ-TuRC alone is thought to be insufficient for it to promote microtubule nucleation; it must also be activated. Purified γ-TuRCs have relatively low nucleating activity, but this increases when they are mixed with fragments of tethering proteins that contain the CM1 domain [27]. When these truncated fragments are expressed in cells, ectopic microtubules are nucleated throughout the cytoplasm [27]. How the binding of CM1-domain proteins induces γ-TuRC activity is not known, but the structure of the budding yeast γ-TuRC suggests that a flexible linker region in GCP3 must move in order to position the γ-tubulin molecules correctly for microtubule assembly [6]. This structure, however, was generated from Spc110-bound γ-TuRCs, suggesting that binding of a CM1-domain protein is not sufficient to move the GCP3 linker, at least in budding yeast. Moreover, artificially inducing structural rearrangements that better positioned the γ-tubulin molecules resulted in only approximately two-fold enhancement of nucleation activity [112]. Thus, other mechanisms must exist, one of which is phosphorylation. Mimicking phosphorylation of *Saccharomyces cerevisiae* Spc110 increases nucleation activity approximately three-fold [84], and NME7 kinase increases the nucleation activity of human γ-TuRCs by approximately 2.5-fold [68]. Whether these phosphorylation events lead to a conformational change in the γ-TuRC, or bring about some other process, is not known. In summary, it is likely that, *in vivo*, a combination of allosteric regulation by CM1-domain proteins and phosphorylation helps to activate γ-TuRCs. It is possible that different activation mechanisms function in different cells and at different MTOCs and how this is regulated remains an exciting area for future research.

## γ-TuRC heterogeneity

**Figure 3:**
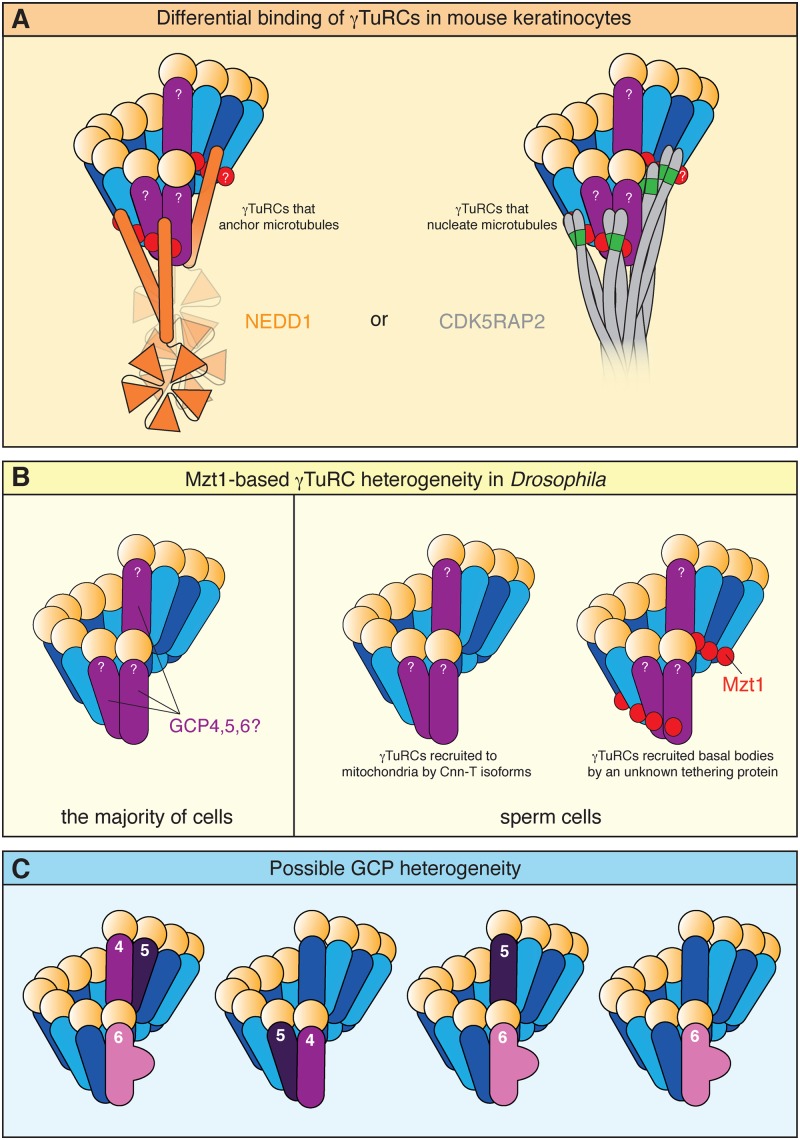
Known and potential forms of γ-TuRC heterogeneity (**A**) In mouse keratinocytes, γ-TuRCs are bound mutually exclusively by either NEDD1 or CDK5RAP2, suggesting that both tethering proteins bind to a similar region of the γ-TuRC. NEDD1-bound γ-TuRCs serve to anchor microtubules while CDK5RAP2-bound γ-TuRCs nucleate microtubules. The position of GCP4, 5 and 6 within γ-TuRCs in these cells remains unknown. (**B**) In *Drosophila*, most γ-TuRCs do not contain MZT1 (red), which is expressed only in the testes. Within the testes, MZT1 is predominantly expressed in sperm cells but in early elongating sperm is only present in γ-TuRCs that are recruited to the basal body, and is not present in γ-TuRCs recruited to mitochondria. The position of GCP4, 5 and 6 within *Drosophila* γ-TuRCs remains unknown. (**C**) While the position of GCP4, 5 and 6 within γ-TuRCs remains unknown, positive FRET data in HeLa cells suggest that GCP4 and GCP5 are adjacent to each other within the ring, while negative FRET data suggest GCP6 is not adjacent to either GCP4 or GCP5 (left) [29]. Stoichiometry measurements from HEK293T cells and immunoprecipitation experiments from HeLa cells suggest that some complexes do not contain GCP6 (middle left) [27] and that some complexes do not contain GCP4 (middle right) [58] respectively. GCP6 can still associate with γ-TuRCs in the absence of GCP4 or GCP5, suggesting that some complexes can form with only GCP6 (right) [28].

All eukaryotes express the core γ-TuRC components γ-tubulin, GCP2 and GCP3, but not necessarily all of the additional components. For example, the *Candida albicans* genome contains only MZT1 in addition to the core components [64], while *Drosophila* additionally contains GCP4, GCP5, GCP6, NEDD1/Grip71, MZT1 and NME7 [5]. The classical view is that all γ-TuRCs within the same organism have the same protein composition, but this has now been disproved. The first evidence came from studies showing that not all mammalian γ-TuRCs contain NEDD1 [27,62]. This led to the suggestion that subpopulations of γ-TuRCs may exist [5]. It was then shown that NEDD1 and CDK5RAP2 bound mutually exclusively to γ-TuRCs in mouse keratinocytes and that NEDD1-bound γ-TuRCs functioned to anchor microtubules while CDK5RAP2-bound γ-TuRCs nucleated microtubules (Figure 3A) [49]. Most recently, it was shown that *Drosophila* MZT1 is expressed only in the testes and is present in, and required for, the γ-TuRCs that are recruited to basal bodies, but not mitochondria, in sperm cells (Figure 3B) [38]. The differential association of Mzt1 with only a subset of γ-TuRCs may also occur in *Arabidopsis thaliana* [62], although this analysis is complicated by the presence of two *MZT1* genes in plants. Collectively, these studies have now shown beyond doubt that γ-TuRC composition can vary between tissues and even between MTOCs within the same cell, and that this can influence γ-TuRC recruitment and function.

What about other γ-TuRC components? Current data does not rule out that they could also confer γ-TuRC heterogeneity (Figure 3C). This can be difficult to test, as experiments typically assay the whole population of γ-TuRCs. For example, while GCP4, 5 or 6 can co-immunoprecipitate with each other in all pairwise combinations from human cell extracts [25], it remains possible that different subsets coexist, e.g. GCP4/5-only complexes, GCP5/6-only complexes and GCP4/6-only complexes. Recent FRET and cross-linking experiments in HeLa cells detected direct interactions between GCP4 and GCP5, suggesting these proteins are adjacent within γ-TuRCs, but the data cannot rule out that these interactions took place in only a subset of complexes [29]. Indeed, a comparison of protein levels after immunoprecipitating GCP6 or γ-tubulin from HeLa cells shows that similar amounts of GCP5 are co-immunoprecipitated but that much less GCP4 is co-immunoprecipitated with GCP6 [58], suggesting that some complexes contain GCP5 and GCP6 but not GCP4. Moreover, GCP6 can localise to SPBs in the absence of GCP4 and GCP5 in both fission yeast [67] and *Aspergillus nidulans* [19] and GCP6-containing γ-TuRCs can be detected in extracts that have been depleted of GCP4 or GCP5 [28], suggesting that GCP6-only complexes can exist. Most data highlight GCP6 as being more important for γ-TuRC assembly than GCP4 or GCP5 [19,28,67], but γ-TuRCs purified from human embryonic kidney cells using CDK5RAP2 fragments contain sub-stoichiometric levels of GCP6, i.e. <1 molecule per γ-TuRC [27], suggesting that not all γ-TuRCs contain GCP6. This may reflect differences in the composition of γ-TuRCs between cell types or between γ-TuRCs bound by different tethering proteins or may simply reflect the difficulty of measuring the stoichiometry of the γ-TuRC components. Clearly more work is required to see whether other types of γ-TuRC heterogeneity really do exist and what functional relevance this might have.

## Other microtubule nucleation factors

**Figure 4:**
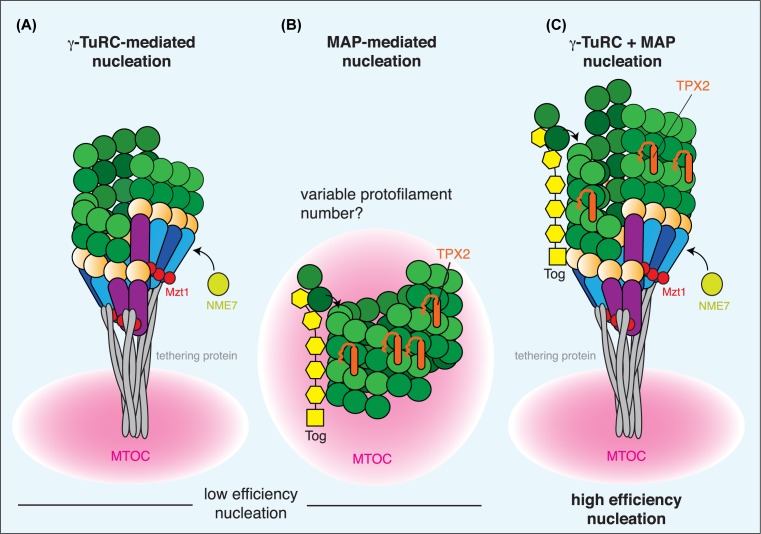
Different modes of microtubule nucleation (**A**) The γ-TuRC templates the addition of tubulin dimers to form a microtubule, but is predicted to promote only lateral and not longitudinal interactions between tubulin dimers. (**B**) Under certain conditions, the combination of tubulin dimers and microtubule-associated proteins (MAPs) is sufficient to promote microtubule nucleation. Tog domain proteins help polymerise the microtubule by promoting the longitudinal addition of tubulin dimers. TPX2 homologues bind across tubulin dimers within the lattice and help prevent catastrophe of the nascent microtubule seed. (**C**) *In vivo*, it is likely that a combination of a γ-TuRC and these MAPs drive highly efficient microtubule nucleation. This presumably occurs at centrosomes, where all of these proteins concentrate, but whether other MTOCs (that are less-efficient microtubule nucleators) use specific mechanisms remains unknown.

It is now becoming clear that two types of non-γ-TuRC proteins can promote microtubule nucleation: Tog domain proteins, such as XMAP215, and homologues of TPX2 [113–117]. Tog domain proteins are microtubule polymerases that regulate microtubule growth by promoting the longitudinal addition of tubulin dimers via interactions between tubulin and the Tog domains, and it is now thought that this also occurs during the early stages of microtubule nucleation [113–118]. Strong evidence suggests that this involves interactions between Tog domain proteins and γ-TuRCs, as budding yeast Stu2 forms a complex with Spc72-bound γ-TuRCs [119], fission yeast Alp14 co-immunoprecipitates with γ-TuRC components [116], and the C-terminal domain of XMAP215 binds γ-tubulin [117]. An elegant model has been proposed [118] in which the γ-TuRC mediates the lateral interactions between tubulin dimers and sets the 13-protofilament lattice structure, while Tog domain proteins, bound to the γ-TuRC via their C-terminal domain, promote the longitudinal addition of tubulin dimers. TPX2 homologues, however, are also important for microtubule nucleation. It was shown that *Xenopus* TPX2 promotes the phosphorylation of NEDD1 by Aurora A to stimulate microtubule nucleation [101], but it has recently emerged that TPX2 homologues also help prevent catastrophe of nascent microtubule seeds [113–115,120]. Recent structural data show that TPX2 proteins bind across longitudinal and lateral tubulin interfaces within the microtubule lattice [121]. Thus, microtubule nucleation is likely most robust in the presence of the γ-TuRC, a Tog domain protein and a TPX2 homologue (Figure 4).

While XMAP215 and TPX2 homologues appear to work synergistically with a template to promote microtubule nucleation [113,114,116,117], there is strong evidence that microtubules can be nucleated both *in vitro* and *in vivo* in the absence of γ-TuRCs [113,115,122–127]. Consistent with this, *in vitro* studies have shown that XMAP215 and TPX2 homologues are sufficient for microtubule nucleation at relatively low tubulin concentrations [113,115], and the Tog domain protein Stu2, but not the γ-TuRC, is required for kinetochore-driven microtubule formation in budding yeast [127]. In contrast, however, XMAP215-mediated nucleation is inefficient in the absence of γ-TuRCs in *Xenopus* egg extracts [117], and Zyg9 and Msps are not required for γ-TuRC-independent microtubule nucleation from centrosomes in *Caenorhabditis elegans* embryos [124] or from acentrosomal sites in cultured *Drosophila* cells [126] respectively. Thus, mechanisms of microtubule nucleation may vary and might be related to the particular cell type or MTOC; it is also possible that other regulators of microtubule nucleation remain to be discovered.

Given that at least some microtubule populations can be nucleated independently of γ-TuRCs, it is worth contemplating why cells need γ-TuRCs at all. We believe there are at least three reasons: firstly, templated microtubule nucleation is more efficient [13,27,49,69,84,87,112–114], presumably as it promotes the lateral interactions between tubulin dimers. Secondly, γ-TuRCs can define the 13 protofilament arrangement found in most cell types, which may (or may not) allow molecular motors a more efficient route along straight protofilaments [128]. Thirdly, γ-TuRCs can help regulate microtubule polarity by defining the position of the minus end. Collectively, these functions presumably explain why γ-TuRCs are essential for cell and organism viability [10,12,16–22].

## The γ-TuRC as a drug target

γ-TuRCs may offer good anticancer targets given their roles during cell division. Currently, some of the most common and most effective chemotherapy agents, such as Taxanes and Vinca Alkaloids, bind microtubules directly. These compounds, however, often lead to a condition known as chemotherapy-induced peripheral neuropathy (CIPN) [129,130]. CIPN presents as numbness, pain, tingling, and heightened sensitivity in the extremities; it limits drug dosage and/or duration and can persist after chemotherapy, and it is a major cause of cancer survivor disability [129,130]. The cellular mechanism by which CIPN occurs is not fully understood, but dying back of axonal projections in the epidermis has been observed in patients and in model systems and this could be caused by axonal transport defects [131–133]; however, alternative mechanisms have been suggested, including mitotoxicity and disruption to calcium homoeostasis [134]. Targeting γ-TuRCs, instead of microtubules directly, may offer a viable alternative [44,135,136] because inhibiting γ-TuRCs would perturb cancer cells [44,135] but may not have a dramatic effect on mature neurons, which would already have generated and stabilised their microtubule networks. For example, axonal transport along stable pre-existing microtubules in neurons might remain unperturbed after γ-TuRC inhibition. Moreover, microtubule severing in neurons may be able to compensate for any reduction in microtubule generation via the γ-TuRC pathway. That said, there is evidence that γ-TuRCs bind to the sides of pre-existing microtubules and regulate microtubule dynamics [125,137], and so it will first be important to assess the role of γ-TuRCs in neurons. The challenge will then be to develop drugs that can inhibit γ-TuRC function in a highly specific manner. Currently, the only γ-TuRC-inhibiting drug is Gatastatin, which was identified by testing derivatives of drugs known to bind α/β-tubulin and was found to bind γ-tubulin with a 12-fold greater affinity than α/β-tubulin [138]. It may also be important, however, to consider γ-TuRC heterogeneity, as this may help increase specificity. Thus, the non-core γ-TuRC components may provide good targets for anticancer drugs, as their inhibition may affect only subsets of γ-TuRCs. Of course, we first need to understand more about γ-TuRC heterogeneity and the role of each γ-TuRC component within the complex.

## Conclusion and perspectives

The combinatorial complexity created by the variety of γ-TuRC components and their various tethering proteins is likely to grant cells the ability to regulate very precisely the assembly, recruitment and activity of their γ-TuRCs. The γ-TuRC has been studied for decades and many key insights have been made. One of the most important developments since the discovery of γ-tubulin [139,140] was the determination of the yeast γ-TuRC structure at near atomic resolution [6]. This finally proved the template model, helped reveal a potentially key step in γ-TuRC activation [112] and, combined with the crystal structure of human GCP4 [15], has changed the way we think about the position and function of the GCP proteins [29]. Clearly, a similar structure of a γ-TuRC from a higher eukaryote would be extremely informative. The more we understand, however, the more questions arise. For example, what causes the flexible region in GCP3 to move in order to position the γ-tubulin molecules correctly? Do all GCP proteins fit into the same γ-TuRC ring? If so, how? If not, how do different GCP proteins affect γ-TuRC behaviour? How do phosphorylation events induce functional changes in the γ-TuRC? How is the binding between γ-TuRCs and γ-TuRC-tethering proteins regulated in a multi-MTOC, multicellular context? These are all exciting questions for the future.

## Summary

- Structural data from yeast has established that the template model for γ-TuRC-mediated microtubule nucleation is correct. In budding yeast, γ-TuRCs comprise a single-turn helical ring of seven γ-TuSCs. In higher eukaryotes, it is likely that GCP4, 5 and 6 replace some of the GCP2/3 molecules within the helical ring. How this occurs, and the precise function of GCP4, 5 and 6, remains unclear.
- Several γ-TuRC components have been discovered only recently. Of these, MOZART1 is the most conserved through evolution and is the best studied, functioning in γ-TuRC recruitment (and possibly γ-TuRC assembly) in several systems.
- γ-TuRCs are recruited to various microtubule organising centres (MTOCs) in cells via γ-TuRC-tethering proteins that normally contain an N-terminal CM1 domain. Binding of these CM1 domain proteins to γ-TuRCs is important for γ-TuRC recruitment, but can also influence γ-TuRC assembly and activation.
- The composition of γ-TuRCs varies between species, but can also vary within the same species and even within the same cell. More work is needed to understand the extent of this heterogeneity and its functional relevance.
- There is an emerging role for non-γ-TuRC proteins in microtubule nucleation. Several recent studies have shown that chTOG domain proteins and TPX2 homologues work synergistically with γ-TuRCs (or artificial templates) for efficient microtubule nucleation.
- γ-TuRCs have been identified as potential anti-cancer targets and the first γ-tubulin inhibitor, gatastatin, has recently been developed. γ-TuRC-inhibiting drugs could in theory lead to a reduction in the occurrence of chemotherapy-induced peripheral neuropathy (CIPN), although this remains to be explored.

## Abbreviations

CIPN: chemotherapy-induced peripheral neuropathy
CM1: centrosomin motif 1
GCP: γ-tubulin complex protein
MTOC: microtubule organising centre
MZT1: MOZART1
MZT2: MOZART2
SPB: spindle pole body
γ-TuRC: γ-tubulin ring complex
γ-TuSC: γ-tubulin small complex
